# Permanently Reprocessable Highly Cross-Linked Thiourethane Networks Derived from Isocyanate-Reactive Amine Catalyst

**DOI:** 10.1021/acsapm.5c04412

**Published:** 2026-02-06

**Authors:** Kailing Lin, Andrew Terentjev, Alessandro Bonifacio, Etienne Piantanida, Eugene M. Terentjev, Mohand O. Saed

**Affiliations:** † Cavendish Laboratory, 2152 University of Cambridge, J. J. Thomson Avenue, Cambridge CB3 0HE, U.K.; ‡ Cambridge Smart Plastics Ltd, 18 Hurrell Rd, Cambridge CB4 3RH, U.K.; § 621186 EssilorLuxottica, Piazzale Luigi Cadorna 3, 20123 Milan, Italy

**Keywords:** vitrimer, thiourethane, covalent adaptable networks, recycling, shape memory

## Abstract

Vitrimers combine thermoset-like stability with thermoplastic-like reprocessability. Thiourethane polymers retain the advantages of classical polyurethanes while enabling efficient dynamic covalent bond exchange. We present a library of high-performance, recyclable thiourethane networks with tunable structure–property relationships and excellent shape-memory behavior. To improve recyclability, we introduce two key strategies: (1) covalently bonding the amine catalyst to an isocyanate group, which prevents catalyst leaching and ensures permanent recyclability; and (2) a reprocessing method that blends ground recycled material with uncured resin, followed by curing and compression under heat to restore mechanical and optical properties. Together, these approaches yield seamless, mechanically robust shape-memory polymer networks and support the sustainable design of smart materials with the potential for multiple life cycles and reduced environmental impact.

## Graphical abstract

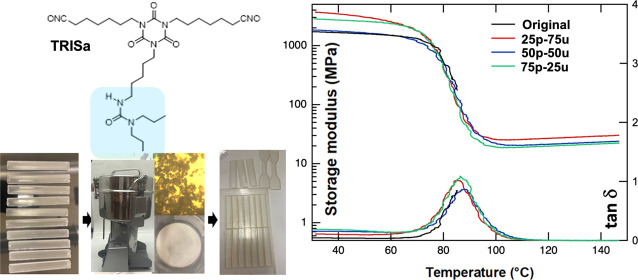

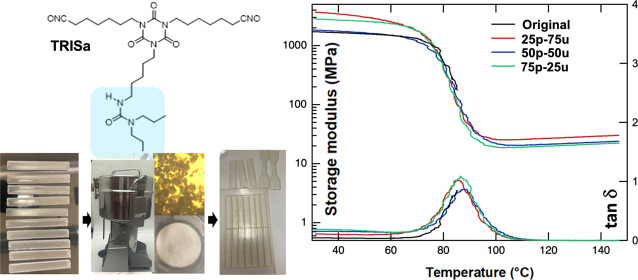

## 1 Introduction

Shape memory polymers (SMPs) exhibit the capability to deform into a temporary shape and retain it below a certain transition temperature but recover the original shape upon exposure to appropriate stimuli, such as heat,^1–4^ electricity,^5^ light,^6^ or magnetic field.^7^ SMPs have been proposed for a range of applications, including biomedical devices,^8,9^ electronic devices,^10–12^ sensors,^13^ and deployable structures.^14,15^ The vast majority of current SMPs are made of elastomers,^16^ hydrogels,^17^ thermoplastic polymers,^18^ or amorphous brittle polymer networks,^19^ which often lack adequate mechanical strength and toughness that are necessary for engineering applications. High-performance thermoset polymers such as epoxies,^20,21^ polyurethanes,^22–24^ polythiourethanes,^25–27^ acrylates,^19,28^ and polyimides^29^ are ideal SMP materials for engineering applications due to their mechanical strength and their thermal and chemical stability. High-performance thermoset SMPs must undergo permanent cross-linking to achieve excellent thermal stability, high mechanical strength, and good chemical resistance. This covalent cross-linking defines the natural shape. However, a permanently cross-linked network renders reprocessability and recycling impossible. Therefore, high-performance thermosets cross-linked with dynamic exchangeable bonds have been utilized to address the processability and recycling challenges while maintaining their mechanical integrity alongside the shape-memory performance.^30,31^

The emergence of a new class of polymeric materials cross-linked with exchangeable bonds has led to the development of covalent adaptable networks (CANs). Bond exchange enables traditional thermoset networks to undergo an elastic–plastic transition at a high temperature and shear stress (although light-activated exchange is also known, e.g., allyl sulfide–thiol exchange),^32^ to enable postpolymerization processing and recycling. Two distinct types of bond-exchange reactions can occur within a CAN network to make it dynamic: a dissociative bond exchange (e.g., Diels–Alder reaction),^33^ when the bonds are made to break and then reform in a different topology, or an associative bond exchange (e.g., transesterification),^34^ when the bonds can only break when the new bond configuration is already available and established, thus never changing the total number of covalent bonds in the material. Both pathways occurring simultaneously are also possible. The term “vitrimer” strictly refers to an associative reaction mechanism.^35–37^ Such materials bridge the gap between thermoplastics and thermosets: they are permanently cross-linked and insoluble in solvents, yet unlike conventional thermosets, they can plastically flow at high temperatures due to bond exchange, enabling reprocessing and true multiuse capability.^34^ This elastic–plastic transition is driven by thermally activated dynamic covalent exchange, fundamentally different from thermoplastics, which become viscous fluids above their melting point.^38^

Chemically cross-linked polythiourethane (PTU) thermosets have been known for a long time and used in applications that require high heat and chemical resistance.^39–41^ PTUs exhibit mechanical properties comparable to those of conventional polyurethane (PU) materials because of their similar chemical structure, where sulfur atoms replace in-chain oxygen. This structural similarity allows hydrogen bonding to occur in a manner just like that of PU. However, PTUs offer several advantages. The formation of PTUs from isocyanates and thiols is characterized as a *click*-type reaction, which mitigates side reactions typically observed in PU synthesis.^42^ Additionally, PTU thermosets possess a higher refractive index due to the presence of sulfur, rendering them promising candidates for optical applications.^43^ Recently, Torkelson, Bowman, and Serra research groups, at the same time, have investigated the two bond-exchange mechanisms acting in thiourethane polymer networks.^44–46^ The dynamic bond of thiourethane linkages can be triggered by organometallic^47,48^ or amine catalysts^46^ or a combination of both.^44^ Amine catalysts offer several advantages over organometallic catalysts. They efficiently catalyze the initial “*click*” reaction between thiol and isocyanate and the reaction during the bond-exchange between thiourethane linkages, resulting in a more homogeneous network formation without the generation of unexpected moieties. Additionally, amine catalysts are less toxic compared to organometallic catalysts.^49,50^ The current method for preparing dynamically cross-linked PTU relies on adding amine catalysts to the monomer mixture of thiol and isocyanate. However, this often results in a rapid and uncontrollable reaction, leading to gelation within a few seconds.^44^ Alternatively, the addition of toxic organometallics, such as dibutyltin dilaurate, has been explored, but these compounds may easily leak from the network or degrade after a few reprocessing cycles.^48^ To address the challenges associated with the rapid catalytic activity of amine catalysts and the toxicity or instability of organometallic catalysts, we propose a novel strategy. This approach involves the permanent, covalent incorporation of an amine catalyst into the polymer network, forming internal catalytic moieties that enable indefinite reprocessability while simultaneously moderating the rate of the initial thiol–isocyanate reaction (Figure 1). Specifically, we covalently reacted dipropylamine (DPA), a secondary amine catalyst, with a trifunctional isocyanate (HDI-trimer) to form stable urea linkages. These urea-containing moieties serve as embedded catalytic sites that mildly catalyze the subsequent reaction between residual isocyanates and thiol groups. During reprocessing, these internal catalysts are reactivated, facilitating network rearrangement under mild conditions. The other novel concept employed in this work is the method of reprocessing the resulting vitrimer PTU during recycling. It is well established that the efficiency of plastic flow above the vitrification temperature depends on the stiffness of the network (i.e., the polymer modulus). While soft elastomeric vitrimers are malleable and readily reformed by plastic flow, the stiff, rigid polymer networks require much slower plastic flow^34,51^ —which is often impractical for reprocessing in standard equipment. Since we are exploring quite stiff PTU polymer networks with high cross-linking density here, their direct melt recompounding in standard industrial conditions is impossible. We developed an alternative approach in which highly cross-linked recycled polymer networks are first ground into a fine powder and then blended with fresh uncured thiol and isocyanate monomers. Building on these two innovations, this study investigates the detailed relationship between the composition and mechanical properties of PTU vitrimers, enabling informed material selection for specific applications. Due to their dense cross-linking, all our materials exhibit pronounced shape-memory behavior when cycled above and below their glass transition temperature (*Tg*).

**Figure 1:**
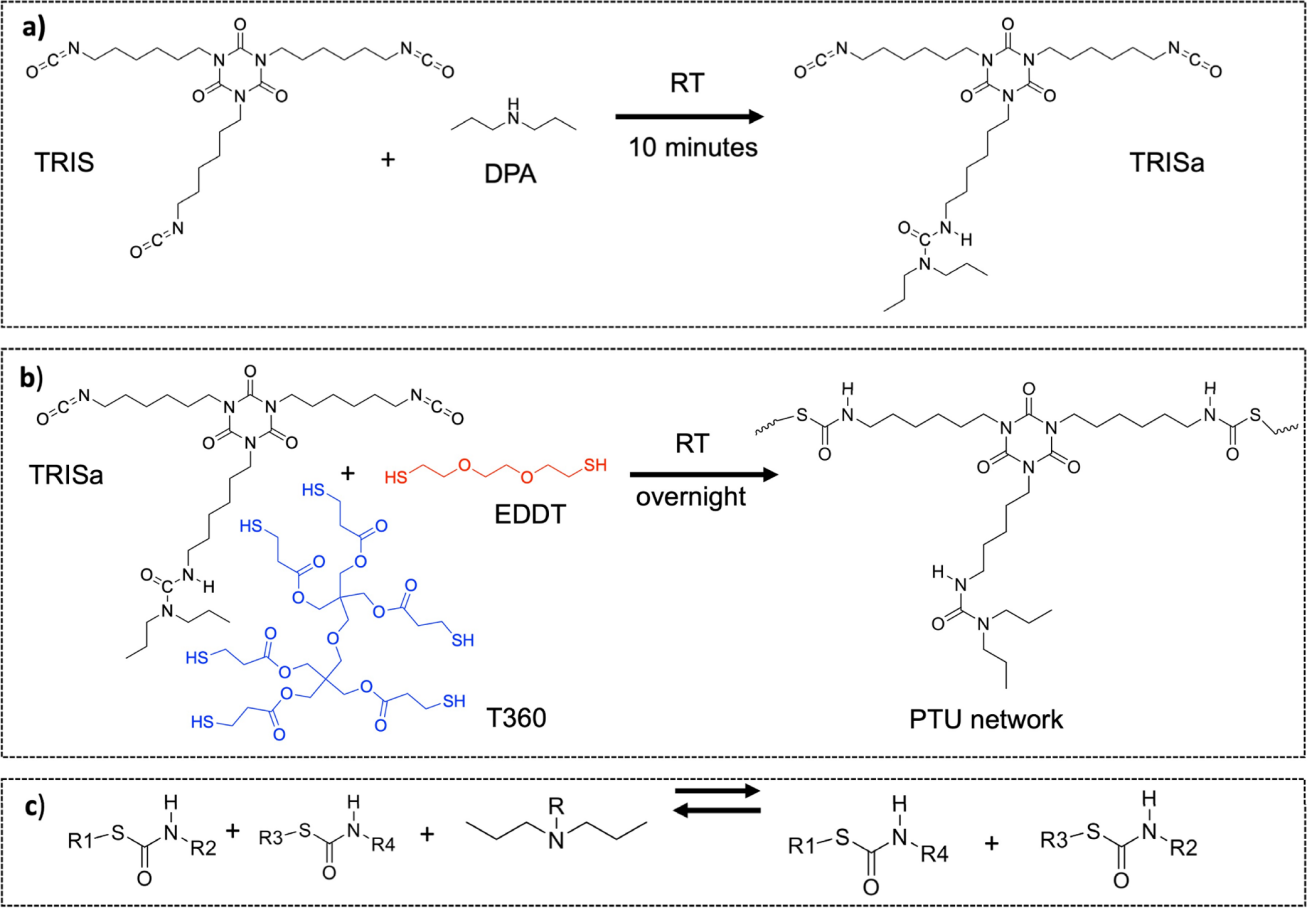
A simplified illustration of the chemical structures and reactions involved in our synthetic process is shown. (a) Rapid room temperature bonding of DPA to TRIS forms a urea linkage through reaction with the tertiary amine in TRISa. (b) Formation of PTU networks using a 1:1 stoichiometric ratio of –NCO to –SH groups, with the mole fraction of T360 and EDDT adjusted to tune network rigidity or ductility. (c) Associative thiourethane bond exchange mechanism in the presence of a tertiary amine catalyst.

## 2 Experimental Section

### 2.1 Materials

2,2’-(Ethylenedioxy)­diethanethiol (EDDT) and catalyst dipropyl amine (DPA) were purchased from Merck-Sigma. Dipentaerythritol Hexakis (3-mercaptopropionate) (thiocure360, T360) was purchased from Tokyo Chemical Industry UK Ltd. 1,3,5-Tris­(6-isocyanatohexyl)-1,3,5-triazinane-2,4,6-trione (TRIS, HDI trimer) was purchased from Biosynth Ltd. All of the starting materials were used as received without any further modification.

### 2.2 Preparation of Poly­(thiol-urethane) (PTU)

The poly­(thiol-urethane) (PTU) networks were polymerized from a trifunctional isocyanate monomer (TRIS), a difunctional thiol spacer (EDDT), and a hexa-functional thiol cross-linker (T360) in the presence of a catalyst (DPA). All molar fractions were calculated with respect to the quantity of isocyanate functional groups, which was taken as 100%. The molar ratios of dithiol to hexa-thiol functional groups were set to 0:100, 5:95, 25:75, 50:50, 75:25, and 100:0, as shown in Table S1. The networks were labeled according to the molar ratio of the hexa-thiol component. The catalyst (DPA) was added at 0.5 wt·% of the total weight of each formulation for all networks. The weight reaction of the monomers is displayed in Table 1.

**Table 1:** Weight Ratio of the PTU Polymer Networks were Measured in Grams

Sample	TRIS (g)	T360 (g)	EDDT (g)
PTU100	1	0.776	0
PTU95	1	0.737	0.027
PTU75	1	0.582	0.135
PTU50	1	0.388	0.271
PTU25	1	0.194	0.406
PTU0	1	0	0.542

A one-pot, two-step amine–isocyanate/thiol–isocyanate reaction sequence was designed to synthesize a library of PTU polymer networks. In the first step, the catalyst (DPA) was added to the trifunctional isocyanate monomer (TRIS) and mixed using a Hauschild SpeedMixer DAC 400 at 2000 rpm for 10 min at room temperature to promote the completion of the amine–isocyanate reaction. In the second step, the difunctional thiol (EDDT) and hexa-functional thiol (T360) were introduced into the same reaction vessel containing the prereacted mixture. The formulation was then mixed in the SpeedMixer at 2000 rpm under a vacuum of 5 bar for 5 min at room temperature. Once a homogeneous and transparent mixture without visible phase separation was obtained, the resulting resin was poured into Teflon molds of various geometries and allowed to polymerize under ambient conditions for 24 h, followed by a postcuring step at 80 °C for an additional 24 h.

### 2.3 Preparation of Reprocess PTU

To demonstrate the reprocessability of PTU polymer networks, PTU95 was selected for recycling and reprocessing due to its highly cross-linked network. The previously prepared PTU95 samples were ground into fine powder using a household food grinder (Philips HL7756, 750 W). The resulting powders were then blended with the uncured PTU95 resin (prepared as described above) using a Hauschild SpeedMixer DAC 400 at 2000 rpm under a vacuum of 5 bar for 5 min at room temperature. The homogeneous mixture of powder and resin was subsequently poured into a metal mold and hot-pressed using a DevilPress system at a pressure of 20 bar and various temperatures, 80, 100, and 120 °C, for 12 h. The weight ratios of ground powder (p) to uncured resin (u) were varied and set to 25:75, 50:50, and 75:25, respectively. The recycled samples were labeled as 25p–75u, 50p–50u, and 75p–25u, where p and u represent the ground powder and uncured resin, respectively.

### 2.4 Characterization

Tensile test: The stress–strain curves of all PTU and recycled PTU samples were obtained using a TiniusOlsen ST1 tensiometer in accordance with ASTM D638–14. The specimens had a width of 6 mm, an effective gauge length of 35 mm, and a thickness of 3 mm. The tests were conducted at an extension rate of 1 mm·min^–1^ under ambient temperature conditions.

Dynamic-mechanical analysis (DMA): DMA was performed using a TA Instruments DMA 850 in tensile film mode on samples with dimensions of approximately 15 × 2.5 × 0.25 mm^3^. A temperature sweep was conducted under an oscillatory tension at a constant frequency of 1 Hz. The storage modulus (*E*′), loss modulus (*E*″), and loss factor (tan δ = *E*″/*E*′) were recorded as functions of temperature from 0 to 120 °C.

Stress relaxation test: The same DMA 850 instrument was used to characterize the stress relaxation behavior of the PTU samples. Specimens with dimensions of approximately 15 × 2.5 × 0.25 mm^3^ were tested in tensile film mode. Each sample was subjected to a constant uniaxial strain of 3%, which was rapidly applied at *t* = 0, and the stress was allowed to relax at the selected temperature until equilibrium was reached, as indicated by the tension force reaching a steady plateau. Prior to strain application, the load-free samples were equilibrated at the target temperature for 5 min.

Iso-stress experiment: “Creep” measurements were performed using a TA Instruments DMA 850 in tensile mode using samples with dimensions of approximately 15 × 2.5 × 0.25 mm^3^. A constant stress of 50, 100, or 200 kPa was applied to the samples. The resulting extensional strain reflected the dynamic Young’s modulus of each network. The strain evolution was subsequently monitored under a temperature ramp of 2 °C·min^–1^, during which the material began to flow upon reaching its characteristic softening temperature.

Shape memory test: The shape-memory behavior of the PTU samples was characterized using a TA Instruments DMA 850 operating in tensile mode, following a five-step procedure: (1) heating: the sample was heated without load above its *T*_*g*_ at a rate of 2 °C·min^–1^ (e.g., for PTU95, *T*_*g*_ = 84 °C; the sample was heated to 100 °C); (2) deformation: at constant temperature, the sample was elongated to a strain of 6% at a strain rate of 1% · min^–1^ to program the temporary shape (shape B).; (3) fixing of shape B: the deformed sample was held at 6% strain and cooled from 100 to 25 °C at a rate of 2 °C·min^–1^ to fix the temporary shape; (4) recovery: the sample was unloaded and reheated from 25 to 100 °C, at 2 °C·min^–1^, during which it recovered its original shape A through spontaneous contraction; and (5) fixing of shape, A: finally, the recovered sample was cooled again from 100 to 25 °C, at 2 °C·min^–1^ under no load. The specimens had dimensions of approximately 15 × 2.5 × 0.25 mm^3^. Throughout the entire procedure, stress, strain, and temperature were continuously recorded as functions of time.

## 3 Results and Discussion

### 3.1 Immobilizing the Amine Catalyst on Isocyanates

Thiourethane chemistry follows the same logic as the ordinary polyurethane, based on the sequential reaction of isocyanates and thiols (instead of diols in PU), and so the established principles of how to obtain the desired material characteristics by optimizing between the soft/flexible and rigid/hydrogen-bonded segments on the chains. The key difference is that thiols lead to the robust “*click*” reaction with no side routes and full conversion.^39,42,50,52^ One of the most effective catalysts for the reaction between –SH and –NCO groups is an amine-based catalyst.^50^ However, there are several downsides to using such catalysts. First of all, small-molecule amine catalysts are often leaching out of the final cured material (amine emissions), which raises the questions of its toxicity in applications.^53^ But even more importantly for our study, where we aim to recycle the polymer networks multiple times, the catalyst must not be allowed to gradually evaporate from the network at high reprocessing temperature because it will be needed to enable the thiourethane bond exchange. Therefore, our first goal is to covalently bind a secondary amine catalyst such as (DPA) to the network while maintaining sufficient mobility to facilitate bonding and exchange reactions in its vicinity. This concept of reacting secondary amines with isocyanates to produce reactive tertiary amine catalysts has previously been applied in the PU industry to stabilize catalysts, thereby enabling low emissions, hydrolytic stability, and improved material compatibility.^53,54^ Figure 1a illustrates the initial step in generating reactive tertiary amine catalysts, by reacting DPA with the trifunctional hexamethylene diisocyanate trimer (TRIS). This reactive tertiary amine catalyst (labeled as TRISa) contains reactive –NCO groups, which can subsequently react with thiol or alcohol groups in traditional PU systems. Different amine catalysts could follow the same principle, but they must involve a secondary amine (as in DPA).^53^ Tertiary amines do not react, whereas primary amines lead to additional cross-linking and reduced mobility of the final bonded catalyst unit. In this work, we used a multifunctional thiol to react with TRISa. The cross-linking density in the final polymer network can be controlled by the mole fraction of hexa-functional thiol (T360) and flexible difunctional thiol (EDDT) (see Figure 1b). The resulting polymer network is covalently bonded to the tertiary amine catalyst, which is vital for extending the lifetime of the polymer networks by enabling permanent recyclability.

Figure S1 (in the Supporting Information) presents the pertinent section of the NMR spectrum of the molecules involved in secondary amine bonding to isocyanate, confirming that at a low mole fraction of DPA, it is fully consumed and does not leave any labile small molecules in the material. It is also important to note that for the subsequent “*click*” reaction with thiol, the rate of reaction is faster with the tertiary amine catalyst than with the secondary amine catalyst. Hence, bonding DPA to isocyanate moieties, thus incorporating them into the network, has this added benefit of allowing us to use even lower concentration of the original DPA catalyst. Therefore, we selected 0.5 wt·% DPA, calculated against the total weight of the final product as an optimal catalyst concentration. Traditionally, 0.8–1.0 wt·% of catalyst has been used; we adopted this lower concentration for the following reasons.^52^ First, the subsequent “*click*” reaction with thiol needs to be not too fast and allow rigorous mixing of components, degassing, and casting into molds while the reacting mixture is still not gelled. On the other hand, if we want to have a strong thiourethane bond exchange at high temperature, there needs to be a sufficient amount of catalyst present in the network. Balancing between these two opposing demands led us to the chosen optimal DPA load in this reaction.

### 3.2 Compositions of Rigid PTU Polymer Networks

Given the relatively low weight fraction of DPA in the initial reaction, most TRIS remains unreacted in the bulk mixture with TRISa, ready for the subsequent “*click*” reaction with thiol. Importantly, the functional bond stoichiometry of 1:1 matching the –NCO and –SH bonds needs to be adjusted to account for the fraction of isocyanate bonds consumed by the DPA. Table 1 lists the weight fractions of the hexa-functional T360 cross-linker and the difunctional flexible EDDT chain extender and the resulting abbreviations of PTU materials reported in this paper (we have tested other combinations as well but selecting these for the clearest presentation). The rationale for this selection is straightforward: PTU100, with the highest cross-linking density, is expected to produce the most rigid polymer, while a gradual increase in flexible EDDT spacers should result in greater ductility. Strictly, if we were to go all the way to zero T360, making a sample of PTU0, we expect to end up with an elastomeric material at ambient conditions—we did not proceed in that direction since this paper focuses on strong structural shape memory polymer networks and their recycling conditions.

Since our polymer is produced via a two-stage reaction, with the first step—bonding of DPA to TRIS isocyanate, our procedure followed the strict protocol. Frist, TRIS and 0.5 wt·% DPA were premixed in a Hauschild SpeedMixer equipped with vacuum control at 2000 rpm for 10 min. T360 and EDDT were then added, followed by a second 10 min mixing cycle under 0.1 bar of vacuum to ensure complete degassing. Polypropylene containers and the dual-axis high-shear mixing under a vacuum provided by the SpeedMixer ensured homogeneous blending. The resulting resin was cast into molds according to the intended characterization: ASTM D638 dogbone specimens for tensile testing, 0.5 mm plates for DMA, and custom molds for shape-memory evaluation.

As explained earlier, the relatively low concentration of amine catalyst (0.5 wt·% of the total mass) ensured that the curing of the thiol–isocyanate resin proceeded slowly enough to provide a practical processing window of approximately 10 min before noticeable gelation. This allowed sufficient time for mixing, degassing, and casting into an open mold without premature curing while still enabling effective bond exchange (see below). In other production settings or with alternative curing methods, a faster curing rate (and thus a higher DPA loading) may be more appropriate, but for our open-mold casting process, this was optimal. Full curing of the thiol-isocyanate resin at such a concentration of tertiary amine catalyst was taking several hours: in our protocol, we left the molds overnight at room temperature and then placed them at 80 °C for 2 h (postcuring) to ensure the completion of all reactions. Even though one should expect some of the bonds to be not able to form in the increasingly solid plastic, none of the monomers could end up labile and leach from the network due to the multiple functionalities of all components.

### 3.3 Mechanical Properties of PTU Polymer Networks

This study establishes a structure–property relationship in rigid PTU networks using two complementary mechanical tests: DMA under small-amplitude oscillation (TA DMA 850, film tensile mode) and tensile testing (Tinius Olsen ST1, ASTM D638). Results are presented in Figure 2 and Table 2. DMA at 1 Hz was employed to determine *T*_*g*_, *E*′, cross-link density (*d*), and the theoretical molecular weight between cross-links (mc). The temperature sweep spanned the glassy state to the rubbery regime. Figure 2a shows *E*′ and tan δ versus temperature, revealing distinct glassy and rubber-elastic plateaus. The tan δ peak shifts systematically with the cross-link density, indicating higher *T*_*g*_ for more densely cross-linked networks. All PTUs remain glassy at ambient conditions with *E*′ ≈ 2 GPa, reflecting their common monomeric composition. Cross-link density was calculated as below:^55^

$$d=\frac{E^{\prime }}{3RT}$$

where *d* (mol·cm^–3^) is the cross-link density, *E*′ (Pa) the rubbery modulus, *R* the gas constant, and *T* the absolute temperature. *E*′ was measured at 130 °C, well above *T*_*g*_. Thermomechanical properties increased with tetrafunctional thiol content: *T*_*g*_ (30–86 °C), *E*′ (7–39 MPa), and *d* (696–3879 mol·m^–3^) from PTU0 to PTU100. This trend corresponds to a decrease in Mc, as calculated using the equation below:

$$M_{c}=\frac{\rho RT}{d}$$

**Figure 2:**
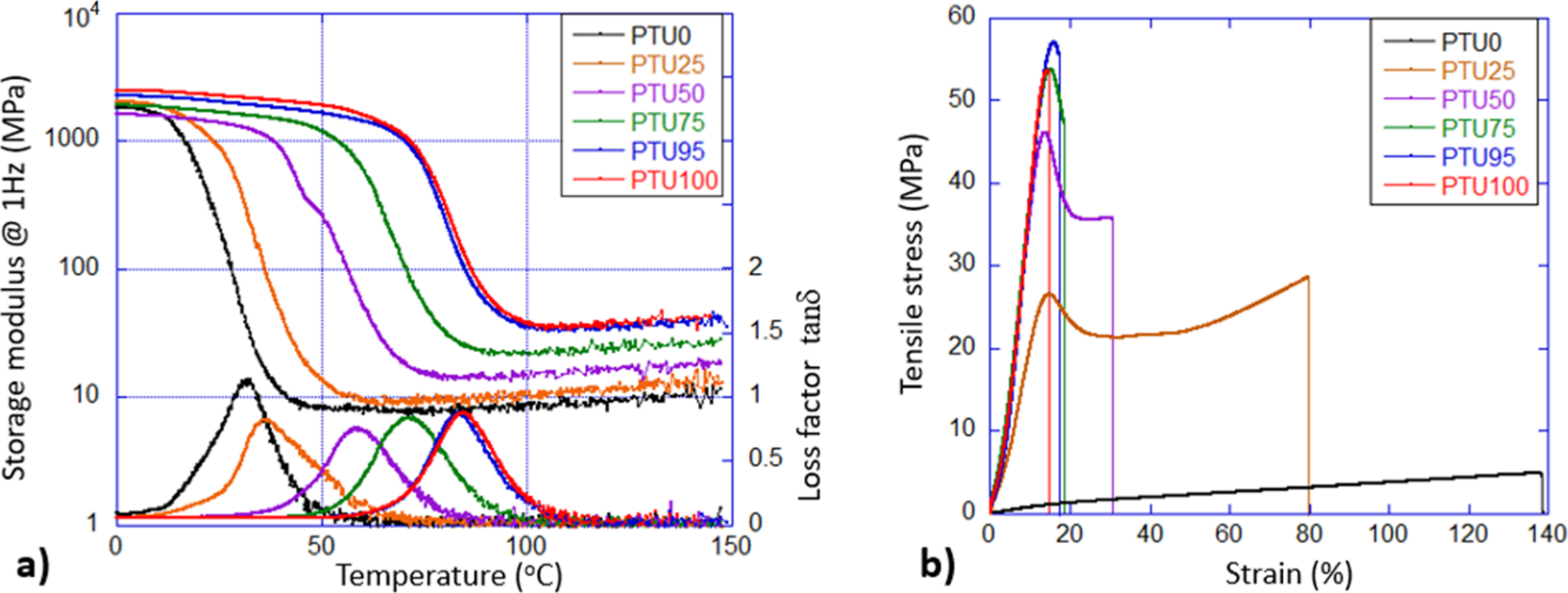
(a) Dynamic-mechanical testing of our PTU polymer networks (samples listed in the plot legend), showing the storage tensile modulus *E*′ (left *y*-axis) and the loss factor tan δ (right *y*-axis the same color), measured at a constant frequency of 1 Hz with increasing temperature. The storage modulus data reveal the glassy regime with *E*′ ≈ 2 GPa and the elastomeric regime where the rubber modulus increases with temperature. The systematic shift of the dynamic glass transition is most evident in the tan δ data. (b) Tensile testing under ASTM D638 standard, showing the systematic increase in ultimate (yield) stress with cross-linking density. The increase in breaking strain for the lower-cross-linked polymers appears more random because crack initiation under stress is a stochastic process influenced by sample imperfections, but the overall trend is clear.

**Table 2:** Thermal and Network Properties of PTU Samples

sample	*T*_*g*_ (°C)	*E*’ (MPa)	d (mol/m^3^)	*M*_*c*_ (g/mol)
PTU100	86	39	3879	283
PTU95	84	36	3580	307
PTU75	74	18	1790	614
PTU50	60	17	1690	651
PTU25	35	13	1292	853
PTU0	30	7	696	1571

*T*_*g*_, rubbery modulus (*E*′), crosslinking density (d), and molecular weight between crosslinks (*M*_*c*_).

where ρ is the polymer density (1.1 g/cm–3). Mc decrees from 1571 g·mol–1 (PTU0) to 283 g·mol–1 (PTU100).

The tensile behavior of PTU polymer networks is shown in Figure 2b. Tests were performed at room temperature using ASTM D638 Type IV dogbone specimens. The stress–strain curves exhibit three key features: the Young’s modulus in the initial linear region, the ultimate tensile stress at the yield point, and the strain at break. Accurate determination of Young’s modulus was limited by minor adjustments in the tensiometer clamps at the start of testing; however, the overall trend of increasing modulus with cross-linking density (relative to the glass transition) is evident. The stress–strain curves reveal a strong dependence of mechanical response on PTU composition (Figure 2b and Table 2). PTU0 exhibits a very low modulus and ultimate stress (∼5 MPa) level but sustains large strains (>130%), indicating a highly compliant, weakly cross-linked network (696 mol/m^3^) with elastomeric behavior (*T*_*g*_ = 30 °C). Introducing more tetrafunctional thiol cross-linker significantly increases stiffness and strength. PTU25 shows a pronounced yield at ∼20–25 MPa followed by strain hardening, reaching ∼30 MPa at ∼80% strain before failure. This behavior suggests a transition from rubber-like elasticity to plastic deformation accompanied by network rearrangement. At higher tetrafunctional thiol cross-linker contents (PTU50, PTU75, PTU95, and PTU100), the materials display high initial modulus and tensile strength, with strength stresses in the range of ∼45–58 MPa occurring at relatively low strains (∼18–20%). These samples fail shortly after yielding, indicating increasingly brittle behavior as the PTU content increases. The sharp stress drop after the peak suggests limited energy dissipation and restricted chain mobility due to increase in the cross-linking density (from 1690 mol/m^3^ for PTU50 to 3879 mol/m^3^ for PTU100). Overall, increasing PTU content shifts the mechanical response from soft, highly extensible elastomers (PTU0) to stiff, high-strength but brittle networks (PTU ≥ 50). These values place PTU networks within the performance range of rigid thermosets and engineering plastics such as epoxy resins and polyurethanes, which typically exhibit tensile strengths of 40–80 MPa and elongations at break of 2–10%.^56^ Despite the relatively low elongation for PTU100, the combination of high tensile strength and moderate ductility, along with hydrogen-bonding interactions, imparts notable toughness compared to conventional brittle thermosets.^26^

### 3.4 Bond Exchange and Stress Relaxation

Thus, it is essential to establish both the conditions and the kinetics of thiourethane bond exchange. One of the most common methods for determining the activation energy of thiourethane bond exchange is stress relaxation testing at various temperatures above *T*_*g*_. Figure 3a presents stress relaxation curves recorded after a rapid strain step (3% strain applied within 3 s) at various constant temperatures during the relaxation period. The theoretical analysis of plastic flow in a vitrimer-like material suggests that there is a single dominant relaxation mechanism, leading to the exponential relaxation law (in great contrast with stress relaxation in thermosets, which is a more complex process of collective relaxation leading to power-law behavior).^38^ Analysis of relaxation curves in Figure 3a gives a very systematic variation of the characteristic relaxation time with temperature—this can be determined from the curve fitted to an exponential law, or equivalently, from the time at which the stress crosses the value 1/*e* = 0.37: there is very little difference between these values, and Figure 3b presents the Arrhenius plot of the logarithm of this relaxation time vs inverse absolute temperature, explored in the activation relationship: Ln­(*t*) *=* Δ*E/k*_*B*_ *T* + const. The slope of this data gives the accurate measure of activation energy that controls the thiourethane bond exchange: Δ*E* = 143 kJ/mol, which is a good match for many other known bond-exchange reactions.^46,57,58^ Note that the same thiourethane associative bond exchange has been studied recently in a soft elastomeric PTU, where significantly faster exchange rates were observed, with activation energies ranging from 50 to 70 kJ/mol.^44,59,60^ This discrepancy arises from two key factors that must be considered in any vitrimer analysis. First, although different catalysts were employed, none were covalently bonded to the polymer chains, resulting in higher mobility and, consequently, greater catalytic activity. Second, it is well established that bond-exchange rates are highly sensitive to network stiffness (i.e., cross-link density). For example, densely cross-linked PTU materials exhibit higher activation energies—often exceeding 180 kJ/mol—due to restricted internal mobility.^48^ In contrast, the softer elastomeric PTU networks allow for more rapid thiourethane exchange due to their lower stiffness.^59^

**Figure 3:**
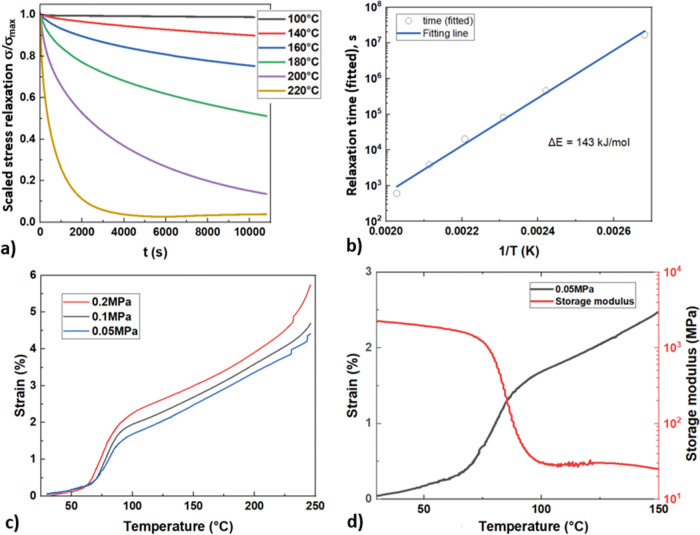
Illustration of stress relaxation in a PTU95 sample, selected to represent one of the stiffest PTU polymers. (a) Curves of tensile stress relaxation after a fast step of 3% strain at *t* = 0, with stress called against the maximum stress at *t* = 0. The characteristic relaxation time is extracted from these curves via exponential fitting. (b) The fitted relaxation time values are plotted on a logarithmic scale against the inverse absolute temperature to enable the Arrhenius fit and the resulting activation energy Δ*E*. (c) “Creep curves” showing the length of the sample under a constant stress and increasing temperature. The creep systematically increases with stress and temperature, until the final sample breaks down at about 250 °C. There is an interesting feature of these creep curves, highlighted in Figure 3d, which shows that the sample softens during the glass transition, before finally settling on the high-temperature creep curve.

Figure 3c,d present the constant-stress creep test: the sample elongation on increasing temperature (at a slow constant rate of 3°/min). As expected, the elongation is greater for higher applied stress, and all samples reach the state of breaking by plastic flow when the temperature reaches about 250 °C. According to the Arrhenius thermal activation analysis above, the stress relaxation time will reach *t* ≈ 100 s at 250 °C; this onset of this plastic background matches the assessment of the rate of this thiourethane bond exchange. An interesting feature, although not central to the main points of our paper, is the rapid increase in the plastic flow (creep) around the glass transition, as illustrated in Figure 3d. The isoforce (at a constant stress of 0.05 MPa) testing shows the creep strain as a function of temperature for PTU95 samples, revealing three distinct regions: (i) slow creep in the glassy region, (ii) a linear and rapid increase in creep around the glass transition region, and (iii) a further increase in creep in the rubbery region. In the glassy region, creep progresses slowly, which is expected due to the restricted mobility of polymer chains. However, the creep rises sharply near *T*_*g*_, driven by enhanced chain mobility and the availability of free volume. Interestingly, the temperature-dependent creep behavior (under isoforce conditions) closely aligns with experimental observations of storage modulus versus temperature and the dynamic glass transition peak in tan δ (see Figure 2a), both coinciding with the sharp rise in creep. Finally, the additional increase in creep within the rubbery region can be attributed to rapid thiourethane bond exchange. In summary, various illustrations presented in Figure 3 establish a clear understanding of the thermally activated bond exchange in these densely cross-linked PTU networks, where the facilitating catalyst is bonded and thus remains at constant uniform concentration across the volume.

### 3.5 Recycling and Reprocessing PTU Polymer Networks

A substantial body of literature on vitrimers and other CANs emphasizes the advantages of reprocessing these covalently cross-linked systems. However, for this concept to achieve practical industrial relevance, reprocessing must be compatible with conventional methods, such as hot-melt compounding and injection molding. In practice, relatively few vitrimers have met this requirement. Starting with Leibler’s foundational work in 2011,^34^ it was shown that densely cross-linked epoxy–acid networks exhibit insufficient flow, even at temperatures well above their vitrification point. Our previous study addressing this challenge concluded that the critical factor is network stiffness (modulus): softer, elastomeric CANs are readily extrudable, whereas rigid CAN networks fail to achieve adequate flow, even at temperatures significantly above their vitrification threshold.^51,59^ By this criterion, the PTU polymer networks examined in this work are highly cross-linked and too stiff to be extruded using standard industrial methods. This limitation is not inherent to the thiourethane bond exchange (or any other dynamic reaction), as the softer, elastomeric variant of the same PTU system has proven fully extrudable and suitable for injection molding.^59,61^ The challenge with dense, stiff networks lies in their low mobility and insufficient bond-exchange rate to enable rapid plastic flow. To address the recycling of highly cross-linked polymers, we developed an alternative reprocessing method inspired by the “self-healing” behavior characteristic of vitrimer rheology. This approach leverages dynamic bond exchange to bridge cracks at the interfaces between polymer fragments. Despite extensive efforts, achieving 100% recyclability of pure PTU plastic proved elusive; recycled PTU typically exhibits reduced mechanical and optical properties, most notably yellow or brown discoloration (see Figure S4 in the Supporting Information). To mitigate this, we ground recycled PTU into a fine powder and thoroughly mixed it with uncured resin, as illustrated in Figure 1b. The resulting resin-powder blend is cured using the original PTU protocol, followed by high-pressure, high-temperature treatment to enhance integration and restore performance. This final step is critical: it activates bond exchange between the recycled PTU grains and the surrounding uncured matrix. Optimization at this stage is essential—higher temperature and pressure improve bonding, but excessive pressure can crack the composite, and excessive heat can cause thermal degradation. After numerous trials (Figure S5), we established optimal conditions at 120 °C and 20 bar compression overnight, producing a seamless, homogeneous recycled plastic. Figure 4 shows the powder obtained after the first grinding stage of the polymer. The particle size is quite heterogeneous (b and c) and could be optimized. While coarse grinding is the easiest option, our tests indicate that the best mechanical properties (comparable to fresh PTU) are achieved when particles are smaller than 50 μm. For this work, we used a basic desktop grinder without filtration, resulting in a mixed particle size distribution, as illustrated in Figure 4b.

**Figure 4:**
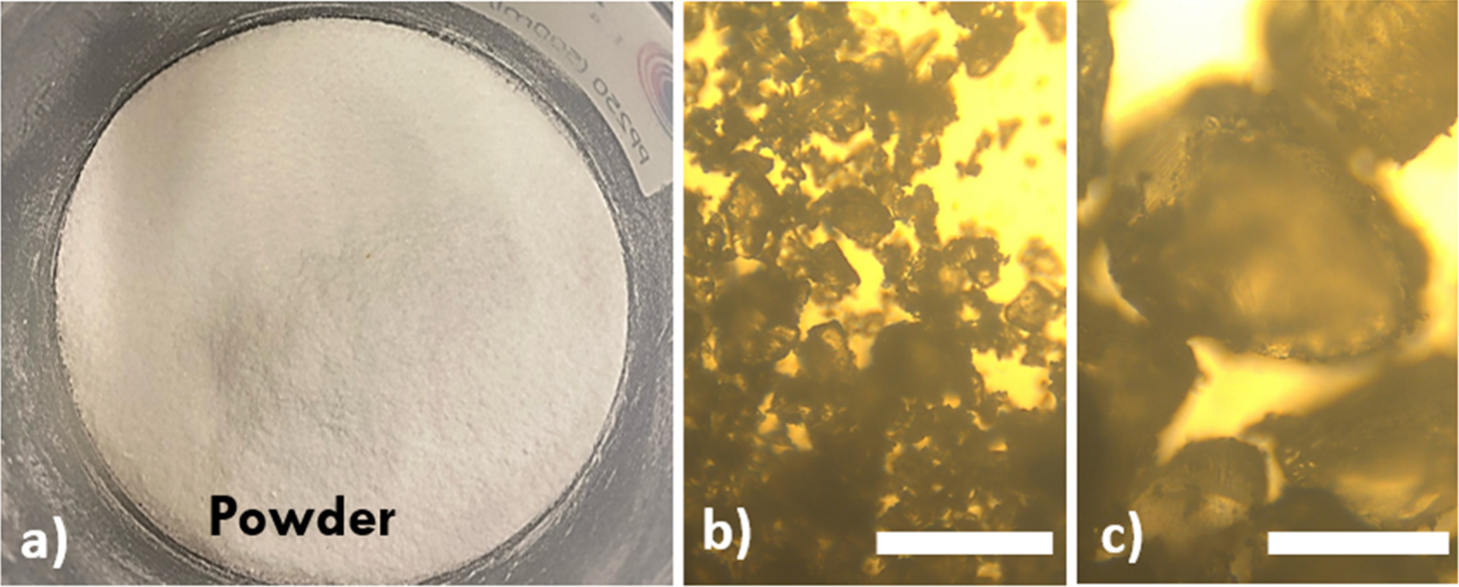
Ground PTU powder: (a) macroscopic view of the bulk powder and (b,c) microscopic images of two representative regions showing particle-size heterogeneity (approximately 10–200 μm). Scale bar: 25 μm.

Mixing the viscous uncured thiol-isocyanate resin with fine polymer powder requires a thorough shearing and vacuum degassing, which we were able to do with the dual-axis Hauschild Speed Mixer, spinning at 2000 rpm for 5 min, under a 5-bar vacuum. After mixing, an opaque honey-like viscous substance was obtained. The mixture was then cast into molds for curing. This process is the similar to the process of curing fresh PTU (leaving overnight at room temperature and then at 120 °C for 2 h). The final stage of postcuring under a heated press produces a homogeneous polymer with no visible traces of powder grains. Figure 5 displays images of three “partially recycled” PTU95 polymer networks with varying recycled powder loadings: (i) 5 wt·% recycled powder and 75 wt·% uncured PTU95 resin, (ii) 50 wt·% recycled powder and 50 wt·% uncured resin, and (iii) 75 wt·% recycled powder and 25 wt·% uncured resin. Notably, the surfaces of these materials are smooth rather than granular, indicating good interfacial bonding and homogeneity even at the highest loading (75 wt·% recycled powder), which represents the practical upper limit for efficient recycling. A slight decrease in optical transparency is observed as the recycled powder content increases, likely due to reduced cross-linking between the powder particles and the uncured resin.

**Figure 5:**
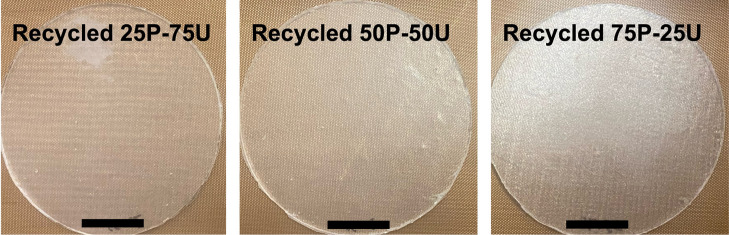
Partially recycled PTU95 samples incorporating different weight fractions of recycled material (as labeled): 25 wt·% recycled powder with 75 wt·% uncured resin (25P–75U), 50 wt·% recycled powder with 50 wt·% uncured resin (50P–50U), and 75 wt·% recycled powder with 25 wt·% uncured resin (75P–25U). Scale bar: 10 mm.

The mechanical properties of partially recycled PTU95 polymer networks compared with those of fresh PTU95 are presented in Figure 6. DMA (Figure 6a) shows that *T*_*g*_ and *E*′ of the recycled formulations exhibit only minor variations relative to those of the fresh material. This consistency can be attributed to the robust dynamic covalent bonding between the PTU powder and the uncured PTU resin, which preserves network integrity even at high recycled content. Furthermore, tensile testing (Figure 6b) confirms that the recycled samples maintain ultimate strength and elongation comparable to those of the original PTU95, demonstrating that multiple recycling cycles do not compromise mechanical performance. Together, these results validate the recyclability of PTU networks without property degradation. It is important to note that samples with higher recycled PTU powder content, such as 75P–25U, were processed under the same recycling conditions (120 °C and 20 bar) as samples with lower powder content. To further investigate processing effects, we varied the recycling temperature (80 °C, 120 °C, and 160 °C) while keeping the pressure constant for 75P–25U samples (see Figure S5 in the Supporting Information). Temperature had a significant impact on the mechanical and optical properties of the recycled polymer. Lower processing temperature (80 °C) adversely affected mechanical performance, with a marked reduction in ultimate strength, modulus, and elongation compared to both unrecycled samples and those recycled at 120 °C. In contrast, higher processing temperature (160 °C) produced mechanical properties comparable to those at 120 °C but resulted in noticeable yellowing, indicating thermal degradation at elevated temperatures. Therefore, 120 °C under 20 bar represents the optimal recycling condition, balancing excellent mechanical performance with the preservation of optical quality while avoiding thermal degradation observed at higher temperatures.

**Figure 6:**
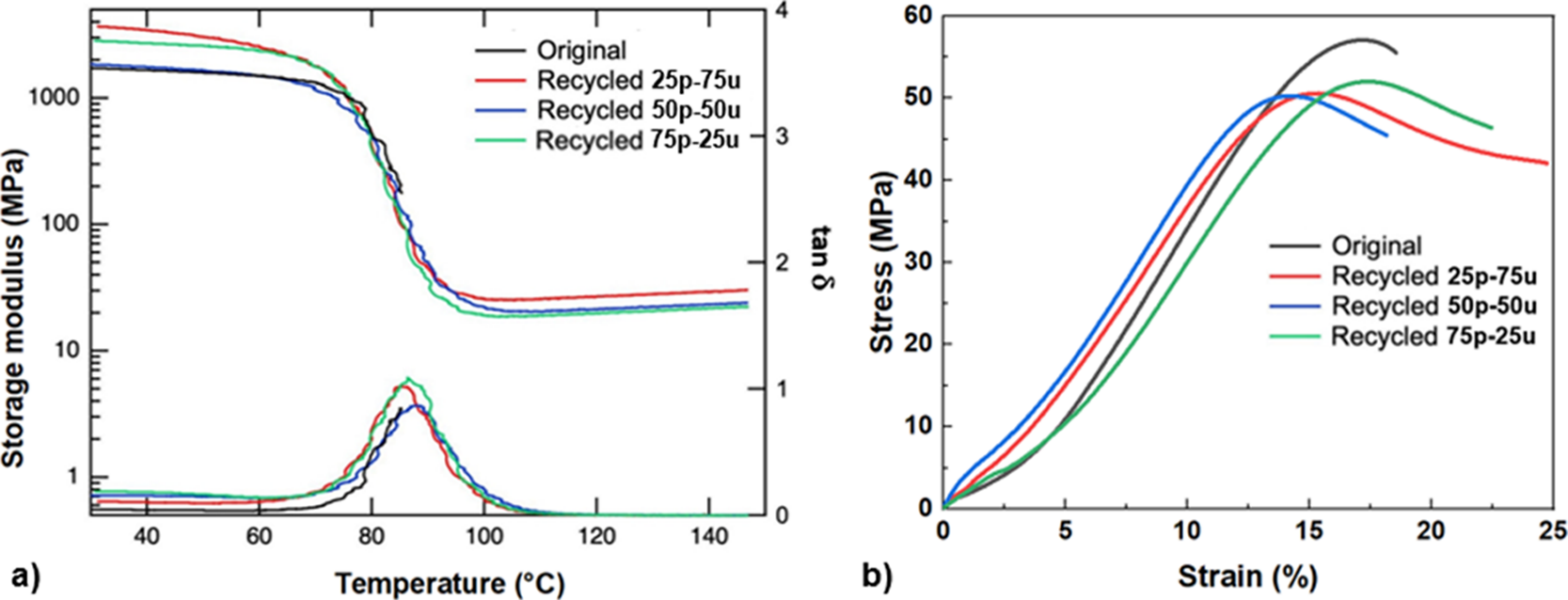
Comparison of the mechanical properties of fresh PTU95 polymer network and three partially recycled variants: 25 wt·% recycled powder with 75 wt·% uncured resin (25P–75U), 50 wt·% recycled powder with 50 wt·% uncured resin (50P–50U), and 75 wt·% recycled powder with 25 wt·% uncured resin (75P–25U). (a) DMA shows no significant difference in either storage modulus or loss factor among the materials. (b) Tensile testing (under ambient conditions) also reveals no measurable difference between the original and recycled samples, confirming that high recycled content does not compromise mechanical integrity.

### 3.6 Shape Memory in PTU Polymer Networks

Cross-linked networks that exhibit either a glass transition or a semicrystalline phase can display the shape-memory effect when thermally cycled around the corresponding transition temperature.^62^ There are various manifestations of this phenomenon. For example, starting with a glassy polymer in equilibrium and having Shape A at ambient temperature, the material can be heated above its glass transition temperature and deformed in the rubbery state into Shape B. Maintaining this shape (i.e., keeping the load) while cooling it back to ambient temperature fixes this deformed state after unloading, as the Shape B′. The difference between Shapes B and B′ is a measure of “fixity”; e.g., if Shape B is exactly equal to Shape B′, then fixity = 100%, which is the case if one does not expect any relaxation in the solid glassy state. If we then take Shape B′ without any load and heat it above the glass transition, it should recover Shape A′, which would be retained on cooling back (still without load) to ambient temperature. The difference between Shapes A and A′ is a measure of “recovery”; e.g., if Shape A is exactly equal to Shape A′,^62^ then recovery = 100%. If there is a plastic creep under load at a high temperature, the recovery will be less than 100%. Without bond exchange, our densely cross-linked PTU polymer networks should have no creep; however, since the vitrimer nature is intrinsic to our polymer networks by the bonded amine catalyst, some creep would always take place.

Figure 7 illustrates this “shape memory” sequence on an example of the 3-point bending test of a rigid PTU50 with its *T*_*g*_ = 70 °C (see Figure 2a). The composite plot is rather complex and requires a detailed explanation. We plot three parameters: the strain on the sample, the corresponding stress, and the temperature, all as a function of continuous elapsed time. There are 5 segments of this test, labeled by “zones” A to E on the time axis. The sample is loaded at zero strain and stress, and ambient *T* = 25 °C. It is then heated at a constant rate to 100 °C without any load (zone A). Here, we register the thermal expansion of the sample, manifesting as increasing strain especially pronounced during the glass transition; this is the same effect we have seen in Figure 3 (c,d). On reaching 100 °C, in the rubbery state of the network (zone B), we apply deformation: the linear strain resulting in the increasing stress. We now fix the strain (sample Shape B) and cool the sample back to ambient temperature (zone C in the plot). The remarkable effect here is the steep rise in stress (at a fixed deformation) as soon as the material enters the glassy state. This is the blocking stress on the cooling sample that attempts to “undo” the earlier thermal expansion, in the high-stiffness glassy phase. Next, on reaching the ambient temperature, we release the load to zero (zone D) and observe the effect of “fixit”’ = 96%. As we heat the unloaded sample back to 100 °C, we expect to see the recovery of the shape the rubbery sample had before deformation inflicted in the zone B. The plot shows the recovery is incomplete; only 82% of the Shape A is recovered, implying that some plastic creep took place during deformation at high temperature. This is not unexpected, since our PTU networks are deliberately designed for bond exchange (and thus recycling), and even though 100 °C is a relatively low temperature for bond exchange (see Figure 3a), a small amount of creep does nevertheless occur under load. Finally, the unloaded sample is cooled back to ambient temperature (zone E in Figure 7), and some thermal contraction occurs again for the sample to finally settle in its final shape. Examination of these steps in a practical experiment exposes various delicate aspects of the overall shape-memory effect, such as inevitable thermal expansion of plastic and the related blocking stress on the attempted contraction under constraint. We shall leave this topic now, referring the interested reader to key literature on this effect.^3,4,62^

**Figure 7:**
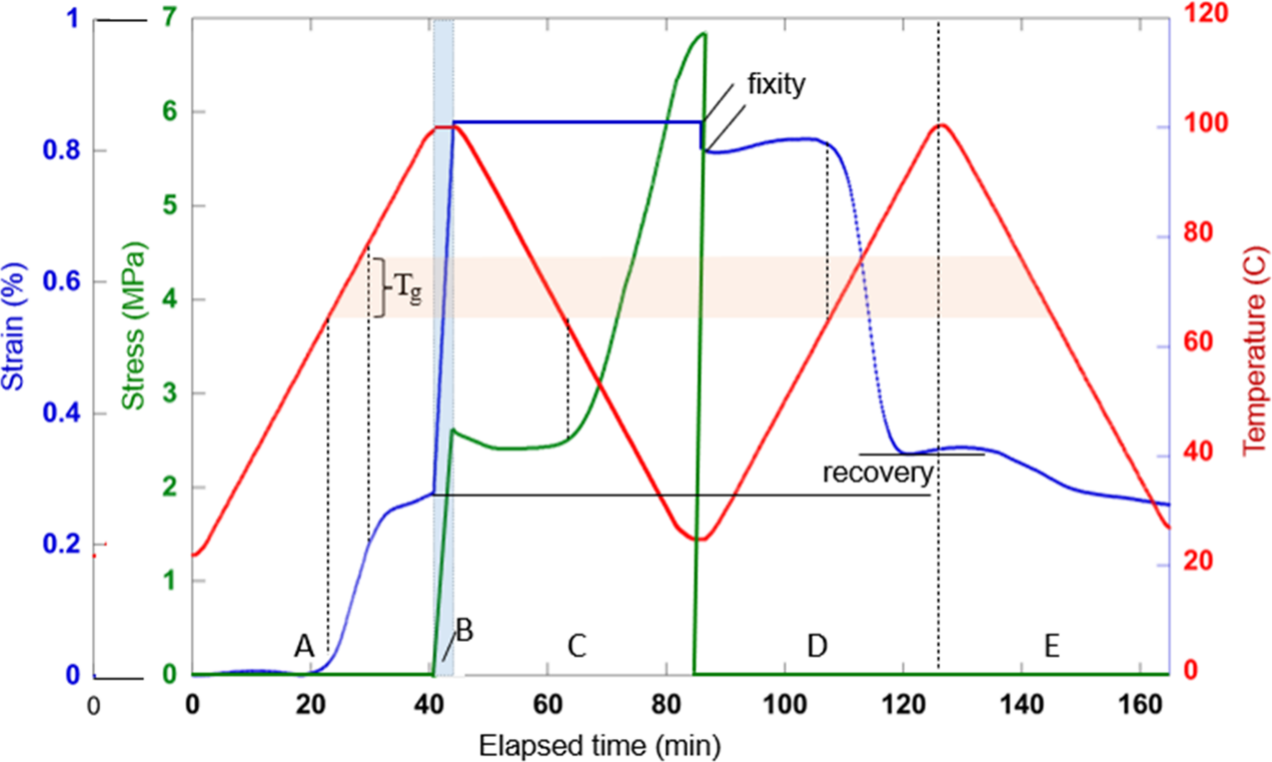
Illustration of the shape-memory cycle during heating and cooling between the rubbery and glassy regimes for PTU50.

For completeness, see the Supporting Information. Figure S7 gives a similar analysis of the shape memory sequence in a tensile test (as opposed to the 3-point bend in Figure 7). The key features are fully reproduced, despite very different deformation magnitudes and geometries, which gives confidence in the analysis presented above. Movie SV2 shows this process of deforming the PTU75 dogbone at 100 °C and “memorizing” its deformed Shape B on cooling—then fully recovering the original dogbone (Shape A) on reheating without load. In these tests, both fixity and recovery were very close to complete. In spite of a certain plastic creep that we detected in the quantitative tests illustrated in Figure 7, the rate of thiourethane exchange at 100 °C is very low, and the time the sample has spent at high temperature under load is very short, so the recovery is close to 100%.

## 4 Conclusion

The overall aim of this study was to develop a permanently recyclable, high-performance thermoset material with a shape memory effect by leveraging thiourethane bond exchange chemistry. To achieve this, we developed a method to retain the necessary catalyst within the polymer network by covalently bonding it into the structure. This approach prevents catalyst leaching and ensures sustained catalytic activity throughout the recycling process. The second innovation introduced in this work is a recycling method for bond-exchangeable rigid thermosets that preserves both mechanical and optical performance. This is accomplished by infusing uncured resin with a recycled PTU material. Since stiff vitrimer materials cannot be directly reprocessed via melt extrusion, we achieved recycling by mixing ground powder from the used plastic with a fraction of uncured thiourethane resin. Remarkably, using just 25% fresh resin (and 75% recycled material) was sufficient to produce a homogeneous new plastic with thermal and mechanical properties indistinguishable from those of the original PTU.

## Supplementary Material







## Notes

Experimental and methods: synthesis, characterization, and recycling methods (PDF)
Polymer processing (MP4)
Shape-memory polymer demonstration (MOV)
