# Supplementary material for: Permanently Reprocessable Highly Cross-Linked Thiourethane Networks Derived from Isocyanate-Reactive Amine Catalyst
Source: ACS Appl Polym Mater. 2026 Feb 6;8(4):3002–13. doi: 10.1021/acsapm.5c04412 (PMC12954761; doi:10.1021/acsapm.5c04412)
Supplement: Supplementary file 1 [file ap5c04412_si_001.pdf]

**Supporting information**

# Permanently Reprocessable Highly Crosslinked Thiourethane Networks Derived from Isocyanate-Reactive Amine Catalyst


Kailing Lin [1,2], Andrew Terentjev [2], Alessandro Bonifacio [3], Etienne Piantanida [3], Eugene M. Terentjev [1,2*], Mohand O. Saed [1,2*]

[1] Cavendish Laboratory, University of Cambridge
J.J. Thomson Avenue, Cambridge, CB3 0HE, United Kingdom

[2] Cambridge Smart Plastics Ltd, 18 Hurrell Rd, Cambridge, CB4 3RH, United Kingdom

[3] EssilorLuxottica, Piazzale Luigi, Cadorna, 3, 20123 Milan, Italy

E-mail: emt1000@cam.ac.uk and mos29@cam.ac.uk


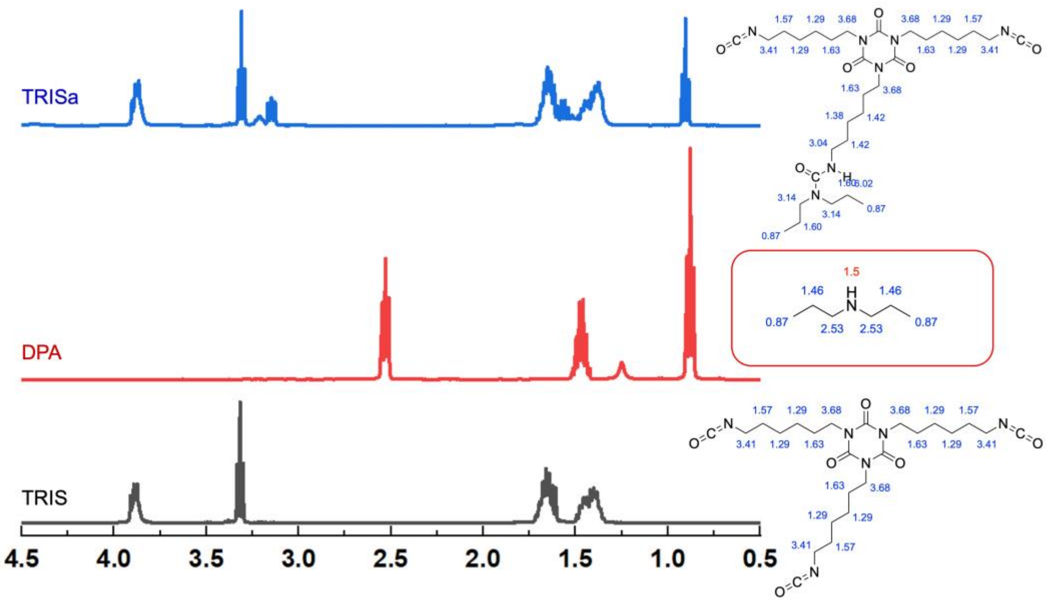


**Figure S1**. NMR spectral evidence of complete bonding of DPA to TRIS: after the reaction shown in Figure 1(a) there is no trace of DPA left in the TRISa product.

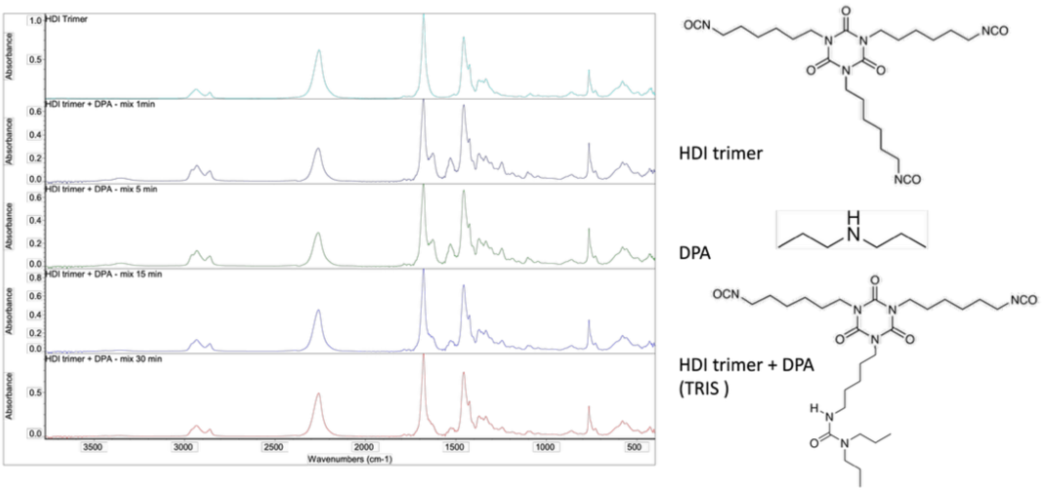


**Figure S2.** FTIR spectral evidence of complete bonding of DPA to TRIS after 30 minutes.

**Table S1.** Compositions, and abbreviated names, of our PTU polymer networks measured in functional molar ratio.

| Sample | TRIS (fuc,mol%) | T360 (fuc,mol %) | EDDT (fuc,mol%) |
|---|---|---|---|
| PTU100 | 1.00 | 1.00 | 0 |
| PTU95 | 1.00 | 0.95 | 0.05 |
| PTU75 | 1.00 | 0.75 | 0.25 |
| PTU50 | 1.00 | 0.50 | 0.50 |
| PTU25 | 1.00 | 0.25 | 0.75 |
| PTU0 | 1.00 | 0 | 1.00 |

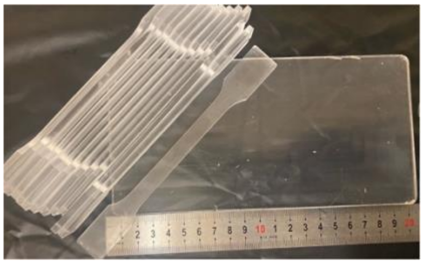

**Figure S3.** Image of cured PTU samples after 24 hours under ambient conditions, followed by a post-curing step at 80 °C overnight

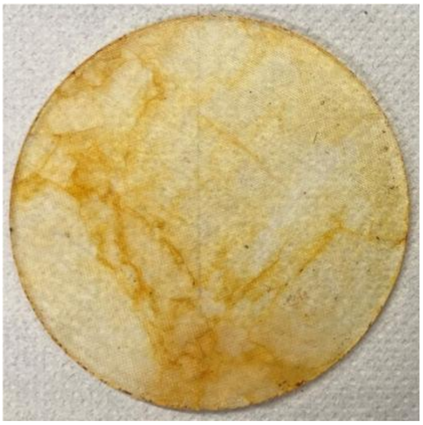

**Figure S4.** Image of recycled PTU at 200 °C under 20 tons of pressure for 30 minutes. The sample exhibits reduced mechanical discoloration, appearing less yellow or brown.

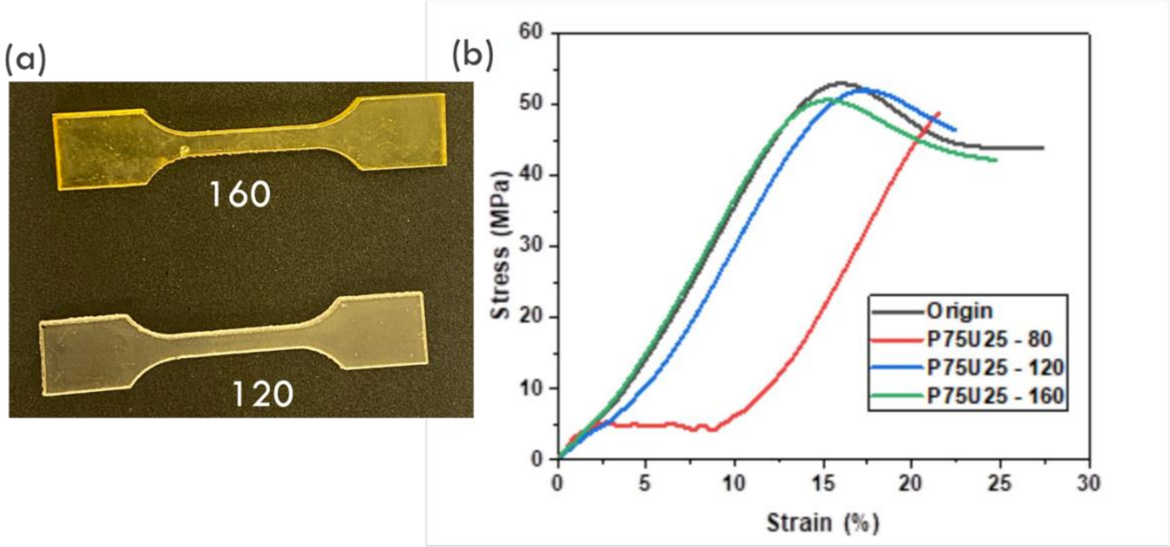


**Figure S5.** Finding the optimum recycling condition for the PTU95 sample, which contains 75 wt% recycled PTU95 powder and 25 wt% uncured PTU95 resin. (a) Image of recycled PTU95 samples processed at 120 °C and 160 °C. (b) Tensile testing (performed by increasing deformation at constant ambient temperature) indicates no significant difference in mechanical performance between the original and recycled materials processed at either 120 °C or 160 °C. The results show that recycling at 120 °C yields samples with superior optical and mechanical properties.

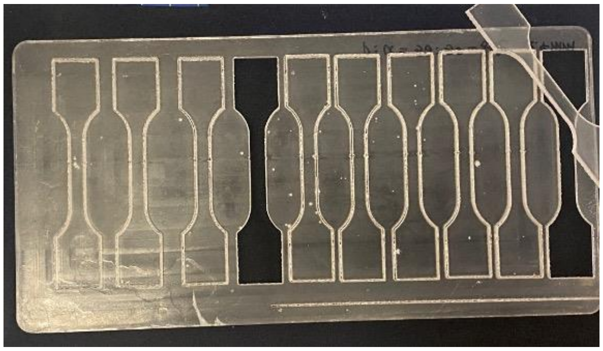

**Figure S6.** An image of recycled PTU95 sample, which contains 50 wt% recycled PTU95 powder and 50 wt% uncured PTU95 resin recycled at 120 °C.

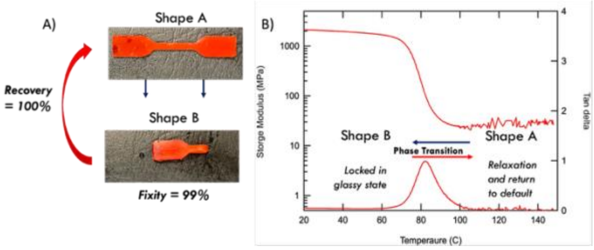


**Figure S7.** illustration of the shape-memory cycle during heating and cooling between the rubbery and glassy regimes.
